# The effect of empagliflozin on circulating endothelial progenitor cells in patients with diabetes and stable coronary artery disease

**DOI:** 10.1186/s12933-024-02466-x

**Published:** 2024-10-28

**Authors:** Roy Hershenson, Inbar Nardi-Agmon, Dorit Leshem-Lev, Ran Kornowski, Alon Eisen

**Affiliations:** 1 Department of Cardiology, Rabin Medical Center, 39 Jabotinsky St., 49100 Petah Tikva, Israel; 2 Faculty of Medicine, Tel Aviv University, Tel Aviv, Israel; 3 Felsenstein Medical Research Center, Rabin Medical Center, Petah Tikva, Israel

**Keywords:** Diabetes and cardiovascular disease, Endothelial progenitor cells, Cardioprotective effects of anti-diabetic medications

## Abstract

**Background:**

Diabetes mellitus (DM) is associated with premature atherosclerotic disease, coronary artery disease (CAD) and chronic heart failure (HF), leading to increased morbidity and mortality. Sodium-Glucose Co-transporter 2 Inhibitors (SGLT2i) exhibit cardioprotective benefits beyond glucose lowering, reducing the risk of major cardiovascular events (MACE) and HF hospitalizations in patients with DM and CAD. Endothelial progenitor cells (EPCs) are bone marrow-derived cells involved in vascular repair, mobilized in response to vascular injury. The number and function of circulating EPCs (cEPCs) are negatively affected by cardiovascular risk factors, including DM. This study aimed to examine the response of cEPCs to SGLT2i treatment in DM patients with stable CAD.

**Methods:**

A prospective single-center study included patients with DM and stable CAD who were started on an SGLT2i (empagliflozin). Peripheral blood samples were collected at baseline, 1 month, and 3 months to evaluate cEPC levels and function by flow cytometry, immunohistochemistry and MTT assays.

**Results:**

Eighteen patients were included in the study (median age 73, (IQR 69, 77) years, 67% male). After 1 month of treatment with empagliflozin, there was no significant change in cEPCs level or function. However, following 3 months of treatment, a significant increase was observed both in cell levels (CD34(+)/VEGFR-2(+): from 0.49% (IQR 0.32, 0.64) to 1.58% (IQR 0.93, 1.82), p = 0.0006; CD133(+)/VEGFR-2(+): from 0.38% (IQR 0.27, 0.6) to 0.82% (IQR 0.7, 1.95), p = 0.0001) and in cell function (from 0.25 CFUs (IQR 0, 0.5) at baseline, to 2 CFUs (IQR 1, 2) at 3 months, p = 0.0012).

**Conclusions:**

Empagliflozin treatment in patients with DM and stable CAD increases cEPC levels and function, implying a cardioprotective mechanism. These findings highlight the potential of SGLT2i in treating cardiovascular diseases, warranting further research to explore these effects and their long-term implications.

**Graphical Abstract:**

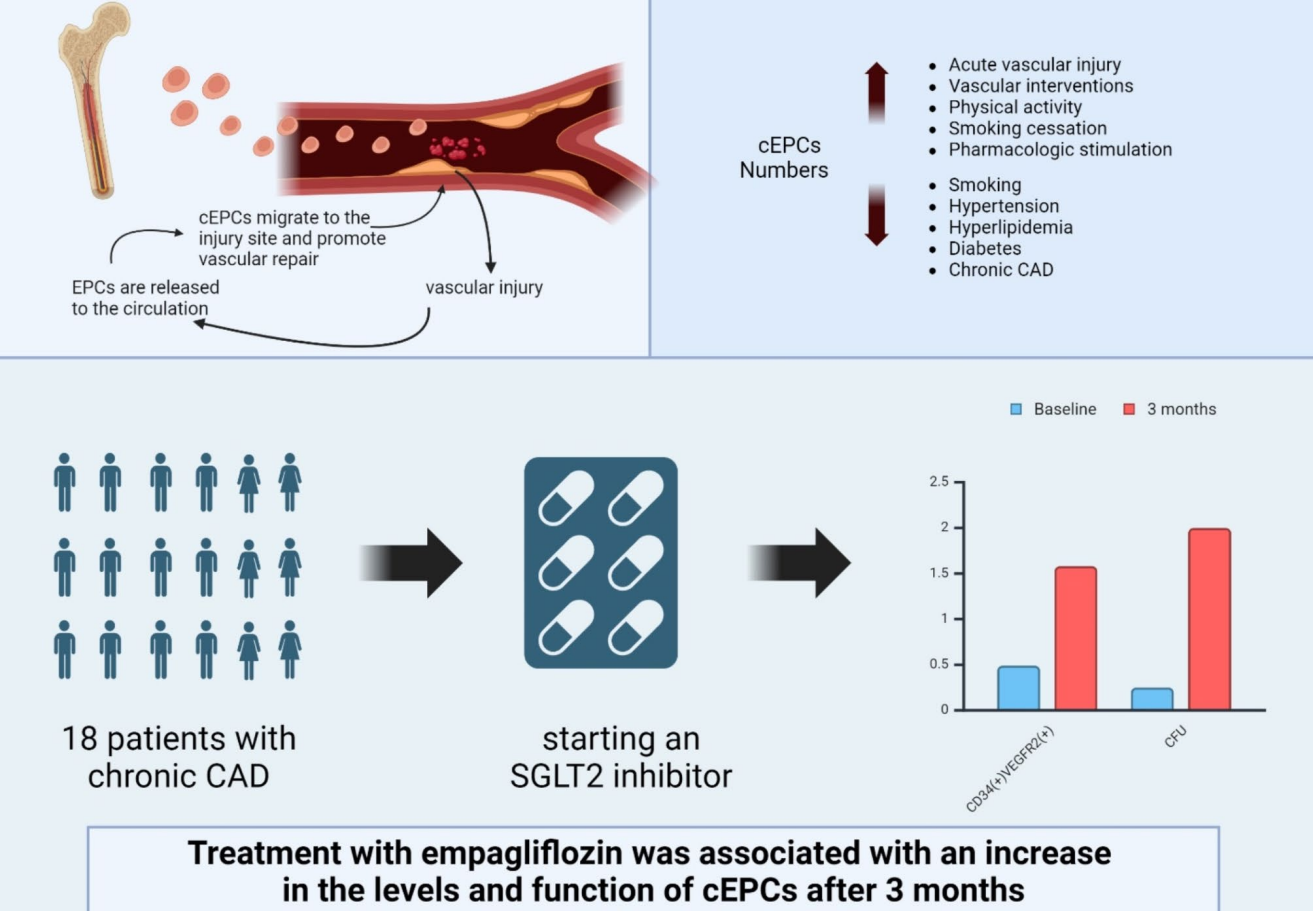

**Supplementary Information:**

The online version contains supplementary material available at 10.1186/s12933-024-02466-x.

## Introduction

Diabetes mellitus (DM) is associated with premature atherosclerotic disease, including coronary artery disease (CAD) and acute coronary syndromes, as well as the development of chronic heart failure (HF), all of which contribute to the increased morbidity and mortality associated with DM [1].

Sodium-Glucose Co-transporter 2 Inhibitors (SGLT2i) are anti-diabetic agents that reduce hyperglycemia by diminishing renal glucose reabsorption in the proximal tubule and increasing urinary glucose excretion [2]. Several clinical trials have demonstrated that SGLT2i have dramatic cardioprotective benefits, including a reduced incidence of cardiovascular (CV) death and HF hospitalizations, in people with and without diabetes and those with and without prevalent heart failure [3–9].^.^

The beneficial CV effects of SGLT2i cannot be attributed solely to their glucose lowering effect as these were observed within a very short time frame and regardless of achieved hemoglobin A1c levels [5, 10]. Moreover, in the DAPA-HF trial the efficacy of dapagliflozin was consistent in those with and without diabetes [5, 8]. In addition to their cardioprotective benefits, SGLT2i are associated with improvement in maximal exercise capacity and quality of life [11].

The mechanisms by which SGLT2i exert their cardiovascular protective effects are not fully understood and several potential explanations, other than glycemic control, have been proposed [2, 5]. Among those are improved cardiac energy metabolism, prevention of adverse cardiac remodeling, diuretic and natriuretic effect, blood pressure reduction, anti-inflammatory and antifibrotic effects, decreasing oxidative stress, prevention of ischemia–reperfusion injury, inhibition of the Na + /H + exchanger, increasing pro-vascular progenitor cells and improving vascular function, and improvement in iron metabolism via reduction in hepcidin, which ameliorates functional iron deficiency and increases myocardial iron content [5, 12–18]. Another suggested theory is that SGLT2i could modify the biology of cells that are involved in the atherosclerotic plaque behavior, such as platelets and endothelial progenitor cells [19].

Endothelial progenitor cells (EPCs) are a unique subtype of circulating, bone marrow derived cells that have the potential to proliferate and differentiate into mature endothelial cells [20]. Different pathological processes, such as acute vascular injury or ischemic damage, trigger mobilization of EPCs to the circulation to promote vascular repair and, in particular, endothelial surface repair, by incorporation into the sites of injury [21–23]. In patients with DM, the number and function of circulating EPCs (cEPCs) are decreased, thus affecting the endothelial repairing mechanism [21, 24, 25].

The mechanisms by which DM is associated with low cEPCs levels remain to be determined. One explanation might be increased apoptosis. Alternatively, DM may interfere with the signaling pathways that regulate EPCs differentiation or mobilization. This reduction, in turn, is associated with an increased risk for development of future CV events including mortality [26–28].

Data regarding the association between SGLT2i and cEPCs levels and function is scarce and equivocal. The aim of this study was to examine the response of cEPCs to treatment initiation with empagliflozin, in patients with DM and stable CAD. We hypothesized that treatment with empagliflozin will be associated with an increase in the levels and function of cEPCs and that the increase in cEPC numbers might constitute an explanation for the beneficial CV effect of SGLT2i.

## Methods

### Study design

A prospective single center study was performed in the cardiology division in Rabin Medical Center (RMC) and the Felsenstein Research Institute.

We recruited patients (age ≥ 18 years) with type 2 DM and stable atherosclerotic CAD [29] that were started on an SGLT2i (empagliflozin) for guideline-based clinical indications [30]. Stable coronary artery disease was defined as known atherosclerotic CAD in asymptomatic outpatients which did not have unstable angina or acute myocardial infarction for at least 3 months prior to their enrollment. CAD was diagnosed by PCI or a non-invasive test. The study was approved by the ethics committee of RMC and informed consent was obtained from all participating patients.

### Study procedures

Blood samples were drawn at pre-specified time points: (1) at baseline (before treatment initiation), (2) 4–6 weeks after the initiation of treatment and (3) 12 weeks after the initiation of treatment with SGLT2i. Samples were evaluated for cEPCs level and function in the basic cardiology laboratory in the Felsenstein Research Center in RMC. cEPC levels and function were compared between the two time points following the initiation of empagliflozin and the baseline (pre-treatment) samples.

All relevant clinical, laboratory, and pharmacological data were extracted retrospectively from patients' electronic records.

### Quantification of cEPC levels

cEPC levels were quantified by measurement of surface markers for immaturity—CD34(+) and endothelial commitment—VEGFR-2(+) using flow cytometry [31]. Although cEPCs have previously been defined as CD34(+)/VEGFR-2(+) cells, as this phenotype identifies cells capable of stimulating angiogenesis in vivo, we also tested the more immature marker of cEPCs CD133(+), which represents a CD34(-)/VEGFR-2(+) subpopulation of precursor cells that can differentiate into CD34(+)/CD133(+) cells under selection pressure favoring endothelial differentiation [32].

Aliquots of mononuclear cells were incubated with monoclonal antibodies against VEGFR-2, CD133 and CD34. Isotype-identical antibodies were used as controls. After incubation cells were washed with phosphate-buffered saline and analyzed with a flow cytometer. Gated CD34 or CD133 positive cells were examined for the expression of VEGFR-2. Results were presented as the percentage of cells co-expressing either VEGFR-2 and CD133, or VEGFR-2 and CD34.

### Functional evaluation of cEPCs

Functional aspects of cEPCs were evaluated by measurement of colony-forming units (CFU) and MTT assay. Levels represent the average of three different repeats at each pre-specified study time point.

*cEPCs CFU quantification:* For the quantification of CFU, isolated peripheral mononuclear cells were re-suspended with Medium 199 supplemented with 20% fetal calf serum. After 48 h, the non-adherent cells were collected and re-plated onto fibronectin-coated 24-well plates (10^6^ cells per well). cEPCs colonies were counted using an inverted microscope 7 days after plating. A cEPCs colony was defined as a cluster of at least 100 flat cells surrounding a cluster of rounded cells. To confirm endothelial cell lineage, indirect immune-staining of randomly selected colonies was performed with antibodies directed against VEGFR-2, CD31 and Tie-2. Results were expressed as the mean number of CFUs per well.

*MTT Cell viability Assay:* The MTT (3-(4,5-dimethylthiazol-2-yl)-2,5-diphenyl tetrazolium bromide) assay is a method used to measure mitochondrial activity in living cells. It operates on the principle that viable cells can reduce MTT, a yellow tetrazolium dye, to formazan, which is a purple precipitate. The amount of formazan produced is directly proportional to the number of living cells, as active mitochondria in viable cells lead to increased MTT reduction. In our study, cEPCs were treated with 1 mg/mL of MTT solution. Following the addition of MTT, the cells were incubated, allowing viable cells to reduce MTT to formazan within the mitochondria. The resultant formazan is then solubilized, and the optical density is measured using a spectrophotometer. The optical density at a specific wavelength is measured, reflecting the amount of formazan produced. Higher optical densities indicate a greater number of viable cells, making this a straightforward way to estimate cell viability and proliferation [33].

### Statistical analysis

The statistical analysis was performed using the R Software. Continuous variables were presented by median and interquartile 25th and 75th range. Categorical variables were presented by (N, %). As study variables were not normally distributed, paired differences for 1 and 3 months, versus baseline, were calculated and the Wilcoxon signed-rank test was used to assess statistical significance. P Values less than 0.05 were considered statistically significant.

## Results

### Patients characteristics

The cohort included 18 patients (median age 73, interquartile range (IQR) 25th, 75th 69, 77 years) with a male predominance (n = 12, 67%). Baseline clinical characteristics are presented in Table 1. Patient-specific information can be found in Tables S1 and S2 (supplements).

**Table 1:** Baseline characteristics of patients before the initiation of empagliflozin

Age in years (IQ1,IQ3)	73 (69, 77)
Male sex (%)	12 (67)
Smoking status (%)	
Never	9 (50)
Past	6 (33)
Active	3 (17)
Hypertension (%)	13 (72)
DM (%)	18 (100)
Mean Hemoglobin A1C% (IQ1,IQ3)	7.47 (7.1, 7.6)
CAD (%)	18 (100)
Prior MI (%)	6 (33)
Prior PCI (%)	15 (83)
Prior CABG (%)	3 (17)
PAD (%)	5 (28)
Prior stroke (%)	1 (5.5)
Baseline medications (%)	
Insulin	4 (22)
Metformin	17 (94)
DPP4i	8 (44)
Repaglinide	1 (5.5)
Aspirin	17 (95)
Clopidogrel/ticagrelor/prasugrel	6 (33)
Anticoagulation (DOACs/Coumadin)	5 (28)
ACEi/ARB	12 (67)
Beta-blockers	11 (61)
Statins	17 (94)
Ezetimibe	7 (39)

Data are presented as median (25th, 75th quartiles) or as percentages, as appropriate. ARB, angiotensin receptor blockers; CABG, coronary artery bypass graft; CAD, coronary artery disease; DM, diabetes mellitus; DOACs, direct oral anticoagulants; ACEi; angiotensin converting enzyme inhibitors; MI, myocardial infarction; PAD, peripheral artery disease; PCI, percutaneous coronary intervention;

### The effect of empagliflozin on cEPC levels

Following 1 month of treatment with empagliflozin there was a numerical increase in the levels of cEPCs that was expressed by an increase of both CD34(+)/VEGFR-2(+) cells, from 0.49% (IQR 0.32, 0.64) at baseline to 0.54% (IQR 0.36, 1.06) and CD133(+)/VEGFR-2(+) cells, from 0.38% (IQR 0.27, 0.6) at baseline, to 0.54% (IQR 0.35, 0.96), but this increment did not achieve a statistical significance (p = 0.092 and p = 0.0593, respectively).

**Fig. 1:**
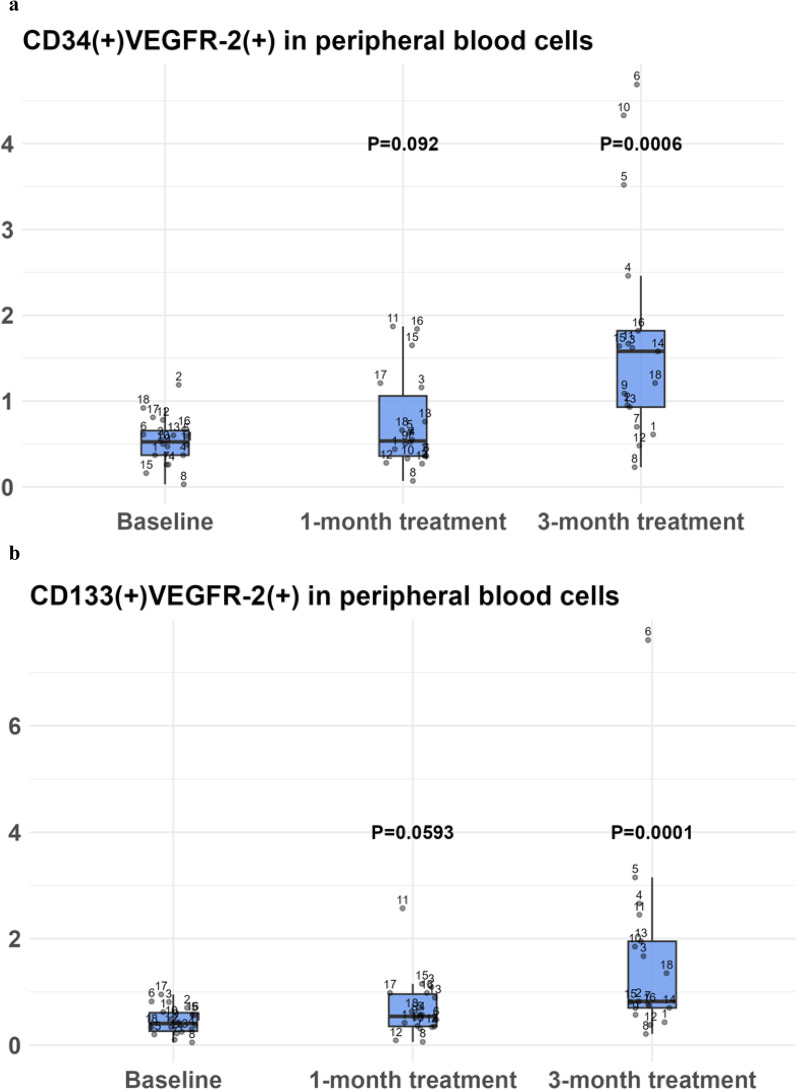
cEPC levels before and following the initiation of empagliflozin. Percentage levels of (**a**) CD34(+)/VEGFR-2(+) and (**b**) CD133(+)/VEGFR-2(+) cells at 1 month and 3 months following the initiation of treatment with empagliflozin. (n = 18). Continuous variables are presented by median and interquartile 25th and 75th range. Categorical variables are presented by (n, %). The Wilcoxon signed-rank test was used to assess statistical significance. Values less than 0.05 were considered statistically significant. *Abbreviations:* cEPC, circulating endothelial progenitor cells; VEGF, vascular endothelial growth factor. *p-values were calculated at 1 or 3-month versus baseline

Following 3 months of treatment there was an increase in the levels of cEPCs, as the CD34(+)/VEGFR-2(+) cell levels were 1.58% (IQR 0.93, 1.82) and the CD133(+)/VEGFR-2(+) cell levels were 0.82% (IQR 0.7, 1.95)- both representing a statistically significant increase compared to baseline (p = 0.0006 and p = 0.0001, respectively) (Fig. 1, Table 2). These findings are compatible with cEPCs activation following the initiation of empagliflozin.

**Table 2:** cEPC levels and function before and following the initiation of empagliflozin

cEPC levels/function at pre-specified time points	Median (IQR 25th, 75th)	p value
CD34(+)VEGFR2(+), T = 0	0.49 (0.32, 0.64)	
CD34(+)VEGFR2(+), T = 1	0.54 (0.36, 1.06)	0.092
CD34(+)VEGFR2(+), T = 2	1.58 (0.93, 1.82)	0.0006
CD133(+)VEGFR2(+), T = 0	0.38 (0.27, 0.6)	
CD133(+)VEGFR2(+), T = 1	0.54 (0.35, 0.96)	0.059
CD133(+)VEGFR2(+), T = 2	0.82 (0.7, 1.95)	0.0001
CFU, T = 0	0.25 (0, 0.5)	
CFU, T = 1	1 (0.5, 1.5)	0.0125
CFU, T = 2	2 (1, 2)	0.0012
MTT, T = 0	0.1 (0.09, 0.11)	
MTT, T = 1	0.13 (0.11, 0.17)	0.004
MTT, T = 2	0.15 (0.13, 0.2)	0.0008

CFU, colony forming units; MTT, 3-(4,5-dimethylthiazol-2-yl)-2,5diphenyltetrazolium bromide; T = 0, levels at baseline (before treatment initiation); T = 1, levels after 1 month of treatment; T = 2, levels after 3 months of treatment

### The effect of empagliflozin on cEPCs function

**Fig. 2:**
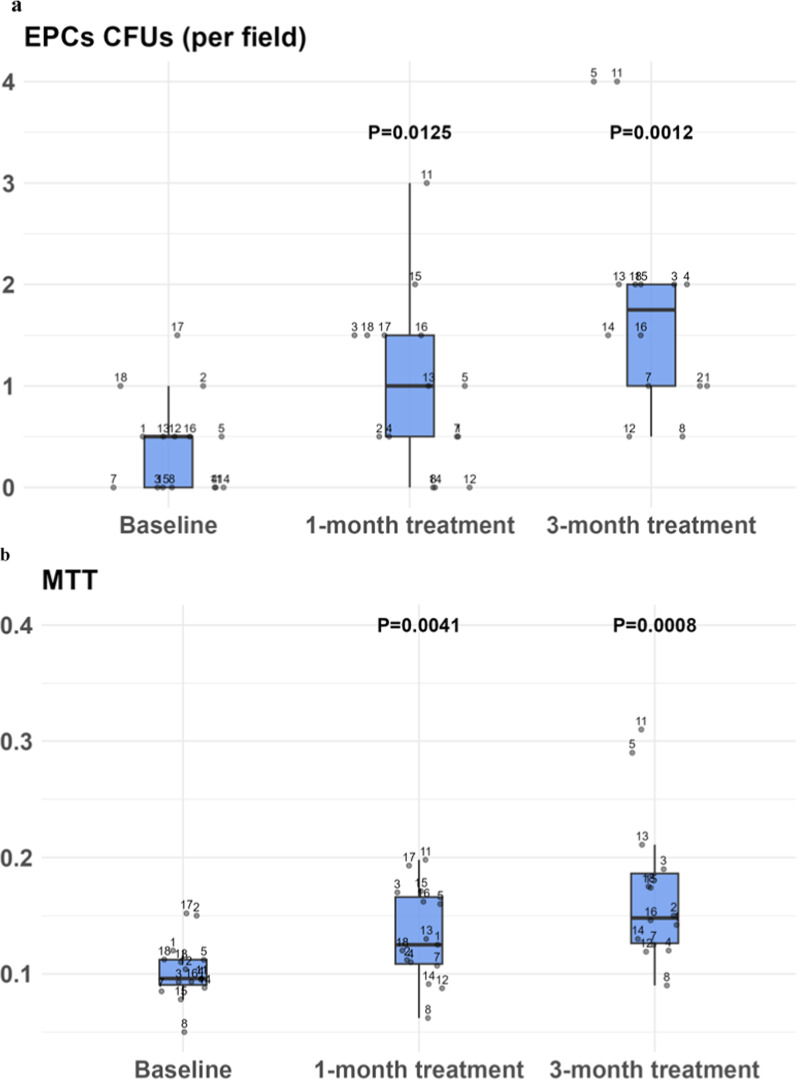
CFUs and MTT assay levels before and following the initiation of empagliflozin. **a** Number of cEPCs-CFUs and (**b**) the results of the MTT assay, at baseline and after 1 month and 3 months of treatment with empagliflozin. (n = 15). Continuous variables are presented by median and interquartile 25th and 75th range. Categorical variables were presented by (n, %). The Wilcoxon signed-rank test was used to assess statistical significance. Values less than 0.05 were considered statistically significant. *Abbreviations:* cEPC, circulating endothelial progenitor cells; CFUs, colony-forming units; MTT, (3-(4,5-dimethylthiazol-2-yl)-2,5diphenyl tetrazolium bromide. * p-values were calculated at 1 or 3-month versus baseline

**Fig. 3:**
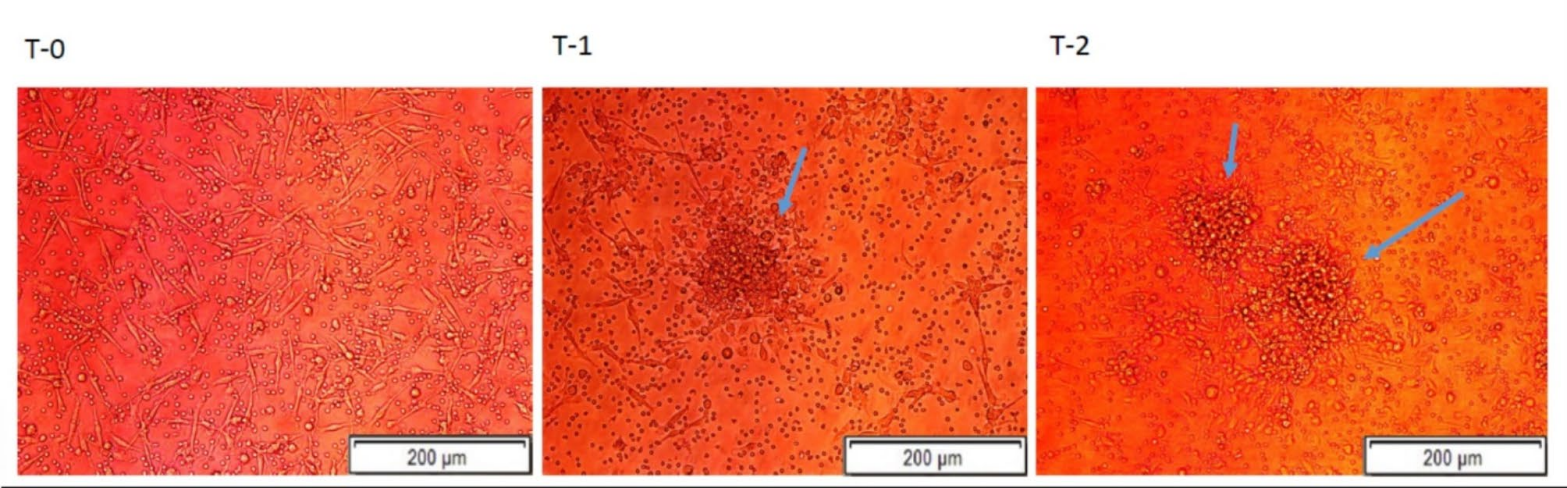
Augmentation of cEPCs CFUs following the initiation of empagliflozin. A representative example showing inverted microscope analysis for cEPCs capacity to form colonies (arrows), before and after 1 month and 3 months of treatment with empagliflozin (patient number 3). *Abbreviations:* cEPCs, circulating endothelial progenitor cells; T0– at baseline; T1– at one month; T2– at three months

Following 1 month of treatment with empagliflozin, functionally active cEPCs were evident microscopically, by their ability to form colonies, from 0.25 CFUs (IQR 0, 0.5) at baseline, to 1 CFUs (IQR 0.5, 1.5) at 1 month, p = 0.0125, and to 2 CFUs (IQR 1, 2) at 3 months, p = 0.0012 (Figs. 2a, 3 and Table 2).

cEPCs viability was evident by an MTT assay which demonstrated elevated levels following treatment initiation, from 0.1 (IQR 0.09, 0.11) to 0.13 (IQR 0.11, 0.17) at 1 month, (p = 0.0041), and to 0.15 (IQR 0.13, 0.2) at 3 months, (p = 0.0008, Fig. 2b). Thus, the increment in functional and viability measures of cEPCs was already noted at one month with additional rise at 3 months' timeframe following empagliflozin initiation.

## Discussion

The results of this study indicate that SGLT2 inhibition with empagliflozin increases the levels and function of cEPCs in patients with stable CAD. The effect of cEPCs was more robust at three versus one month following the SGLT2i initiation. The effect of empagliflozin on cEPCs was not solely related to metabolic control as HbA1C, total cholesterol and LDL-C levels did not change significantly during the follow up period. These findings may reflect a potential mechanism whereby this anti-diabetic medication exerts its CV protective effect.

### The role of EPCs in CAD

EPCs are a diverse group of bone marrow-derived cells that resemble embryonic angioblasts at various stages of development. They play a crucial role in maintaining the homeostasis of the adult endothelium [20, 34, 35]. In healthy vessels, EPCs balance the natural process of apoptosis [36], while in pathologic states of endothelial injuries, the EPCs replace damaged endothelium and participate in neovascularization [37]. When triggered by vascular injury, these cells are released into the circulation and arrive at the area of vascular damage. There, they incorporate into the injured epithelium and stimulate the secretion of growth factors that activate adjacent cells, promoting re-vascularization and vascular remodeling [38]. Consequently, the quantity of circulating EPCs (cEPCs) might influence the body’s ability to repair vascular damage and be a marker of vascular health. Depletion or an impaired mobilization of cEPCs contributes to endothelial dysfunction, the progression of atherosclerotic disease, and increased risk of cardiovascular events [20, 39].

The number of cEPCs have been shown to be reduced in patients with established CAD [40], and reduced cEPCs levels have been found to be an independent predictor of CAD progression [41]. A systematic review and meta-analysis of 9 studies including 4,612 patients on cEPCs in patients who had PCI found the lower baseline EPC count was associated with a significantly greater occurrence of in-stent restenosis (HR 1.33; 95% CI 0.97–1.82, P = 0.045) [42]. In addition, while cEPCs are increased after an acute vascular event [43] and even after coronary interventions [42] cEPCs numbers have been shown to be reduced in patients with chronic stable CAD [44].

### Cardiovascular risk factors and EPCs

Continuous exposure to cardiovascular risk factors can repeatedly damage the vascular endothelium, requiring constant repair and replacement. Mounting evidence indicate that major cardiovascular risk factors interfere with different aspects of EPC biology (mobilization, homing, differentiation and function [45] and the numbers and function of cEPC levels were shown to be reduced in patients with the same cardiovascular risk factors, such as hypertension, hyperlipidemia, diabetes and smoking [38, 45–47].

On the contrary, positive changes in cardiovascular risk factors and lifestyle modifications, such as smoking cessation and increased physical activity, have been shown to result in increased numbers of EPCs [47–49]. Various cardio-protective drugs that were associated with increased EPC count include angiotensin receptor II inhibitors [43, 44], statins [40], PCSK9 inhibitors [50] and others [39–52]. Pharmacologic stimulation of EPCs was suggested to promote repair of endothelial injury and prevent progression of atherosclerosis [39].

### DM and EPCs

Hyperglycemic status activates multiple maladaptive signaling pathways involving endothelial dysfunction, which leads to initiation and progression of atherosclerotic disease. Indeed, cardiovascular disease is the predominant cause of death in diabetic patients, making the prevention and control of cardiovascular disease in patients with DM crucial [1].

In DM, impairment and reduction of cEPCs characterize both type 1 and type 2 diabetes, hindering the conservation of healthy endothelium and promoting endothelial dysfunction and accelerated atherosclerosis [53, 54]. cEPC levels were suggested as biomarkers of diabetes-related cardiovascular complication [54]. Given the negative effect of DM on EPCs, various studies evaluated the change in numbers or function of EPCs in response to anti-diabetic medications other than SGLT2i. Drugs that were shown to increase the number of EPCs include insulin [54–57], sulfonylurea [54–58], metformin [59, 60], thiazolidinediones, [54, 61, 62] DPP-4 inhibitors [54, 63, 64], and GLP-1 receptor agonists [54, 65, 66].

Numerous studies have consistently demonstrated the protective effects of SGLT2 inhibition against cardiovascular disease [3, 6, 9, 67]. The observation that these benefits are achievable within a short time frame and likely independent of glycemic control [3, 68–70] led to studying of other potential effects of SGLT2i, beyond their glucose-lowering properties [69].

SGLT2 proteins are part of the sodium-glucose co-transporter system, responsible for transporting glucose from the renal tubule lumen into renal tubule epithelial cells, thereby facilitating glucose reabsorption from the urine. In the kidneys, SGLT2 inhibition reduces glucose reabsorption and promotes its excretion in the urine [69].

In the cardiovascular system, research has focused on the anti-atherosclerotic properties of SGLT2i, and multiple mechanisms for their CV protective effect were proposed. For instance, treatment with SGLT2i significantly inhibited the migration and proliferation of vascular smooth muscle cells (VSMCs) in vitro [71] and in diabetic patients [72]. Substantial evidence indicates that SGLT2i improve endothelial function [73–78], improves arterial stiffness and vascular smooth muscle dysfunction [76] and reduced oxidative stress [79, 80]. Empagliflozin improved the MicroRNA signature of endothelial dysfunction in patients with DM and HF with preserved ejection fraction [81]. It was also shown to improve flow mediated dilation (FMD) in patients with T2DM [82]. Dapagliflozin also improved endothelial function as assessed by FMD in patients with inadequately controlled T2DM, suggesting a class effect [78]. The addition of evocolumab, a proprotein convertase subtilisin/kexin type 9 (PCSK9) inhibitor on top of empagliflozin, further improved FMD [83].

Other studies have shown that SGLT2i reduced macrophage activity, another inflammatory factor in the atherosclerotic plaque [84–86], and were able to reduce tissue inflammation [87–93]. The reduced vascular inflammatory signaling observed with canagliflozin was attributed to inhibition of IL-1β-stimulated secretion of the key pro-inflammatory, pro-atherogenic mediators in an AMPK-dependent manner [86]. Moreover, treatment with empagliflozin [94] and canagliflozin protected the heart from inflammation via AMPK activation following myocardial ischemia reperfusion injury (IRI) [80, 94]. Empagliflozin has been shown to attenuate cardiac microvascular IRI in human coronary artery endothelial cells through improving mitochondrial homeostasis [95]. Although it was demonstrated that SGLT2i exert anti-inflammatory and anti-oxidant actions in human myeloid angiogenic cells (MAC), empagliflozin did not reverse lipotoxicity induced impairment in MAC, thereby suggesting that its protective effect is unlikely to be mediated through changes in bioenergetic metabolism [96].

SGLT2i attenuated endothelial to mesenchymal transition and cardiac fibroblast activation, suggesting an effect on non-myocyte CV cells [97].

Another suggested theory for their CV protective effect is that SGLT2i could modify the biology of other cells that are involved in the atherosclerotic plaque behavior, such as platelets levels [98] and function and EPCs [99].

Data regarding the association between SGLT2i and cEPCs levels and function is scarce and have been so far equivocal. Bonora et al. [100] did not find any significant change in cEPC levels after 12 weeks of treatment with an SGLT2i, but cEPC levels significantly increased after 1.5 years of treatment. The authors therefore concluded that the increase in cEPC levels was attributed to the improved glycemic control and not to a direct effect of the cells. Similarly, we observed a non-significant change in cEPC number and function at 1-month. However, there was already a trend seen at this early time point, which became more apparent and statistically significant after 3 months. These observations suggest at least some extent of direct effect on the cEPCs, aside from the glycemic control. Nandula et al. showed no significant change in cEPCs levels after 16 weeks of treatment with Canagliflozin, but there was a significant increase in the functionality and migratory response of cEPCs [101]. It is important to note that in both trials treatment with SGLT2i was given to patients who were already receiving baseline treatment with oral anti-diabetic medications and/or insulin that are known to increase cEPCs numbers. Therefore, it can be speculated that cEPC levels were already increased at baseline and therefore their levels did not increase during follow up. Moreover, Bonora et al. did not examine the functionality and migratory response of cEPCs to treatment with SGLT2i [100].

The thought that the observed effect of SGLT2i on cEPCs may be attributed to secondary effects regardless of glucose lowering is supported by our findings that glycemic control, as measured by HbA1c, was within recommended levels at baseline and there was no significant change throughout the study in all patients. Similarly, BP and lipid profile were also within target levels at baseline and did not change during the study period (Table S2).

These observations align with findings from similar studies on cEPCs under cardioprotective medications, which demonstrated an increase in cEPCs independent of the drug's primary effect. For instance, a study by Pelliccia et al., comparing telmisartan with placebo, revealed a statistically significant increase in cEPCs despite no significant difference in the reduction of BP between the groups [44].

*Limitations.* A main limitation of this study is the small number of patients, which led to relatively weak statistical significance. Additionally, data regarding CFUs was missing in three patients. Moreover, the methods used to assess EPCs lack standardization, which may have influenced our results [102]. All patients were treated with empagliflozin, which was the most common SGLT2i used at that time in patients with DM. Thus, we cannot conclude whether other SGLT2i such as dapagliflozin have a similar effect on cEPCs. In addition, the limited follow-up period of 3 months does not allow us to draw conclusions about the long-term effects of empagliflozin on cEPCs.

We were unable to determine whether the increase in cEPCs was due to enhanced migration from the bone marrow or stimulation of EPC proliferation. Furthermore, it remains unclear whether the elevated number of cEPCs had a therapeutic effect on damaged endothelium.

It could be argued that the lack of a control group is another limitation. However, in this study design, the results are based on the mean delta change in each patient at different time points (baseline, 1-month, and 3-month), with the baseline sample serving as a control.

Another limitation is that body mass index (BMI) was not available. Although obesity is inversely correlated with cEPC levels and function [103, 104], weight is not expected to change significantly within 3 months of study.

Lastly, while some baseline medications have been shown to influence cEPC numbers it is unlikely that this impacted the results. Baseline medications were part of chronic treatment regimens maintained over a prolonged period, with no changes made throughout the study (Table S1).

#### Future perspectives

The preliminary results of our study, suggesting an increase in the number and functionality of cEPCs following treatment with an SGLT2 inhibitor, may have important future clinical implications in the treatment of cardiovascular diseases. For example, since cEPCs play a role in the process of neovascularization and may contribute to plaque stabilization, SGLT2i might have clinical relevance in the treatment of acute vascular events such as acute coronary syndrome, acute ischemic stroke, and acute limb ischemia. Additionally, they could have a beneficial role in secondary prevention after ischemic stroke. There is clearly a need for further expanded research regarding these possibilities, as clinical implications of our findings will rely on long-term clinical trials designed to examine clinical endpoints.

## Conclusion

Patients with DM and stable CAD who were treated with empagliflozin showed an increase in both the levels and function of cEPCs over a 3-month treatment period. These findings suggest another cardioprotective mechanism of SGLT2i that extends beyond their glucose-lowering effects. Future studies are necessary to confirm these findings and to explore whether SGLT2i might also be beneficial in other clinical scenarios involving vascular injuries, where an increase in cEPCs levels could provide therapeutic benefits.

## Electronic supplementary material

Below is the link to the electronic supplementary material.

Supplementary Material 1

## Data Availability

All data supporting the findings of this study are available within the paper and its Supplementary Information

## Abbreviations

CAD: Coronary artery disease
cEPC: Circulating endothelial progenitor cells
CFU: Colony forming units
DM: Diabetes mellitus
EPC: Endothelial progenitor cells
HbA1C: Hemoglobin A1C
HF: Heart failure
LDL-C: Low density-lipoprotein cholesterol
MAC: Myeloid angiogenic cells
MACE: Major adverse cardiovascular events
SGLT2i: Sodium-Glucose Co-transporter 2 Inhibitors
