# Supplementary material for: The effect of empagliflozin on circulating endothelial progenitor cells in patients with diabetes and stable coronary artery disease
Source: Cardiovasc Diabetol. 2024 Oct 28;23:386. doi: 10.1186/s12933-024-02466-x (PMC11520434; doi:10.1186/s12933-024-02466-x)
Supplement: Supplementary file 1 — Supplementary Material 1 [file 12933_2024_2466_MOESM1_ESM.docx]

**Table S1. Patients' baseline characteristics**

| **Pt n.** | **Age** | **Sex** | **Smoking**  **Status*** | **Baseline comorbidities** | | | | | | | **Baseline medications** | | | |
| --- | --- | --- | --- | --- | --- | --- | --- | --- | --- | --- | --- | --- | --- | --- |
|  |  |  |  | **HTN** | **Dlp** | **DM**  **(y since diagnosis)** | **IHD**  **(y since diagnosis, y since last PCI)** | **HFrEF (most recent LVEF%)** | **AF/PVD** | **Ischemic stroke** | **Cardiac** | **Anti- DM** | **Anti dlp** | **Anti platelets/ anti coagulation** |
| **1** | 71 | M | Never | - | V | V (8) | V (7;7) | V (40%) | - | - | ACE/ARB  BB | Metformin | statins | Anti platelets |
| **2** | 68 | M | Never | V | V | V (30) | V (0.5; 0.5) | - (60%) | - | - | BB | Metformin  DPP4i  Insulin | Statins  Ezetrol | Anti platelets |
| **3** | 77 | F | Never | - | V | V (10) | V (8;8) | - (45%) | V | - | - | Metformin  DPP4i | Statins  Ezetrol | anti coagulation |
| **4** | 74 | M | Past | V | - | V (12) | V (0.5; 0.5) | - (55%) | - | - | CCB  ACE/ARB  BB | Metformin | Statins | Anti platelets |
| **5** | 79 | F | Current | V | V | V (30) | V (3;3) | - (45%) | - | - | ACE/ARB  Furosemide  BB | DPP4i  Insulin | Statins  Ezetrol | Anti platelets |
| **6** | 71 | F | Past | V | - | V (4) | V (0.5; 0.5) | - (60%) | - | - | CCB | Metformin | Statins | Anti platelets |
| **7** | 76 | F | Current | V | V | V (15) | V (11;6) | - (65%) | V | - | CCB  ACE/ARB | Metformin  DPP4i | Statins | Anti platelets  anti coagulation |
| **8** | 81 | M | Never | V | V | V (4) | V (1;1) | - (65%) | - | - | ACE/ARB  BB | Metformin  DPP4i | Statins | Anti platelets |
| **9** | 69 | M | Past | - | V | V (10) | V (1;1) | - 60%) | - | - | BB | Metformin | Statins Ezetrol | Anti platelets |
| **10** | 58 | M | Past | V | V | V (17) | V (0.5;0.5) | - (65%) | - | - | ACE/ARB | Metformin | Statins  Ezetrol | Anti platelets |
| **11** | 82 | M | Never | V | - | V (8) | V (24;24) | V (25%) | - | - | CCB  ACE/ARB  Thiazide  BB | Metformin | Statins | Anti platelets |
| **12** | 73 | F | Never | V | V | V (15) | V (12;9) | - (60%) | - | - | CCB | Metformin  Insulin | Statins | Anti platelets |
| **13** | 74 | M | Never | V | - | V (14) | V (22;11) | - (60%) | - | - | ACE/ARB | Metformin | Statins | Anti platelets |
| **14** | 69 | M | Past | - | - | V (3) | V (1;1) | - (55%) | V | - | ACE/ARB  BB | Metformin  DPP4i | Statins | Anti platelets  anti coagulation |
| **15** | 63 | F | Never | V | - | V (6) | V (0.5;0.5) | - (65%) | - | - | ACE/ARB  BB | Metformin  DPP4i | Statins | Anti platelets |
| **16** | 72 | M | Current | - | V | V (13) | V (24;24) | - (60%) | - | - | CCB  ACE/ARB  BB | Metformin | Statins  Ezetrol | Anti platelets |
| **17** | 82 | M | Never | V | V | V (17) | V (22;22) | - (55%) | V | - | CCB  ACE/ARB  BB | Metformin  DPP4i | Ezetrol | Anti platelets  anti coagulation |
| **18** | 65 | M | Past | V | V | V (20) | V (1;1) | - (55%) | V | V | CCB | Metformin  Insulin | statins | Anti platelets  anti coagulation |

ACE angiotensin converting enzyme inhibitors; AF; atrial fibrillation; ARB, angiotensin receptor blockers; BB, beta blockers; CCB, calcium channel blockers; Dlp, dyslipidemia; DM, diabetes mellitus; DPP4i,dipeptidyl peptidase IV inhibitor ; HFrEF, heart failure with reduced ejection fraction; HTN, hypertension; IHD, ischemic heart disease; LVEF, left ventricular ejection fraction; PCI, percutaneous coronary intervention, PVD – peripheral vascular disease

**Table S2. Patients' diabetes, cholesterol, blood pressure (BP) and hemoglobin (Hb) values at baseline and at 3 months.**

| **Pt n.** | **Baseline** | | | | | **3 months** | | | | |  |  |  |  |  |
| --- | --- | --- | --- | --- | --- | --- | --- | --- | --- | --- | --- | --- | --- | --- | --- |
|  | **total cholesterol (ng/dL)** | **LDL (ng/dL)** | **Hb A1C (%)** | **BP (mmHg)** | **Hb**  **(g/dL)** | **total cholesterol (ng/dL)** | **LDL (ng/dL)** | **Hb A1C (%)** | **BP (mmHg)** | **Hb**  **(g/dL)** |  |  |  |  |  |
| **1** | 134 | 64 | 6.8 | 130/90 | 13.5 | N/A | N/A | N/A | N/A | N/A |  |  |  |  |  |
| **2** | 122 | 55 | 10.1 | 150/70 | 12.4 | 138 | 60 | 8.7 | 157/72 | 13.7 |  |  |  |  |  |
| **3** | 140 | 75 | 6.9 | 140/65 | 10.4 | 131 | 71 | 6.9 | 143/65 | 9.6 |  |  |  |  |  |
| **4** | 107 | 43 | 7.4 | 145/77 | `13.7 | 109 | 46 | 7.3 | 136/60 | 14.2 |  |  |  |  |  |
| **5** | 100 | 77 | 7.5 | 110/46 | 10.6 | 99 | 73 | 7.1 | 104/60 | 11 |  |  |  |  |  |
| **6** | 102 | 47 | 6.3 | 130/60 | 12.6 | 86 | 52 | 6 | 137/63 | 12.9 |  |  |  |  |  |
| **7** | 136 | 50 | 7.1 | 130/70 | 13.7 | 121 | 55 | 6 | 117/58 | 10.5 |  |  |  |  |  |
| **8** | 155 | 74 | 7.1 | 140/70 | 13.4 | 145 | 69 | 6.6 | 134/78 | 13.1 |  |  |  |  |  |
| **9** | 98 | 48 | 8 | 136/52 | 12.3 | 98 | 47 | 6.5 | N/A | 13.2 |  |  |  |  |  |
| **10** | 145 | 74 | 7.1 | 130/70 | 12.7 | 84 | 33 | 8.8 | 130/80 | 14.2 |  |  |  |  |  |
| **11** | 126 | 59 | 6.4 | 124/73 | 14.5 | 148 | 66 | 6.2 | 112/64 | 15.1 |  |  |  |  |  |
| **12** | 128 | 33 | 8.8 | 148/77 | 10.5 | 120 | 40 | 7.6 | 124/66 | 10 |  |  |  |  |  |
| **13** | 153 | 90 | 7.1 | 140/80 | 15.5 | 148 | 90 | 6.5 | 140/80 | 16.1 |  |  |  |  |  |
| **14** | 114 | 65 | 7.1 | 140/67 | 14.3 | 117 | 58 | 7.4 | 120/66 | 14.7 |  |  |  |  |  |
| **15** | 117 | 49 | 7.5 | N/A | 12.5 | 136 | 72 | 7.1 | N/A | 13.2 |  |  |  |  |  |
| **16** | 144 | 50 | 7.2 | 137/70 | 14.5 | 142 | 78 | 7.2 | N/A | 15 |  |  |  |  |  |
| **17** | 256 | 130 | 7.7 | N/A | 11.7 | 264 | 159 | 7.5 | N/A | 11 | **Δ change after 3 month** | | | | |
| **18** | 252 | 178 | 8.8 | 120/84 | 11 | 125 | 94 | 11.9 | 150/65 | 12.6 | **total choleste-rol (ng/dL)** | **LDL (ng/dL)** | **Hb A1C (%)** | **BP (mmHg)** | **Hb**  **(g/dL)** |
| **Mean**  **(±SD)** | 140  (±44) | 71.5  (±37) | 7.47  (±0.88) | SBP: 134(±10)  DBP:  70(±10) | 12.7  ±1.5 | 130  (±37.5)_ | 66.7  (±22.5) | 7.37  ±1.4 | SBP: 133(±10)  DBP:  68(±7.3) | 12.9  ±1.8 | -10 | -4.8 | -0.1 | SBP: -1  DBP: -2 | +0.2 |
